# 

**Published:** 1910-10

**Authors:** 


					﻿\t*: leiepamy ana leiegrapny.
Vol. XXVI.
OCTOBER, 1910.
No. 4.
T E X A Ss
MEDICAL
JOURNAL
“Red Back”
PUBLISHED AT AUSTIN, TEXAS, BY
F. E. DANIEL, M. D„ - - - -
PRINTED BY THE VON BOECKM ANN-JONES Colr*^‘®’3R£<
X es nir
Subscription, 81.00 a Year, in Advance. -	-	-	- Single Copies, 10 Cents
Entered at the Postoffice at Austin, Texas, as second class mail matter.
				

